# An analysis of research priority-setting at the World Health Organization – how mapping to a standard template allows for comparison between research priority-setting approaches

**DOI:** 10.1186/s12961-018-0391-0

**Published:** 2018-11-29

**Authors:** R. F. Terry, E. Charles, B. Purdy, A. Sanford

**Affiliations:** 0000000121633745 grid.3575.4 TDR, the Special Programme for Research and Training in Tropical Diseases, World Health Organization, 20 avenue Appia, 1211 Geneva 27, Switzerland

**Keywords:** Research priority-setting methods, Database, Standards, Research priority mapping, WHO

## Abstract

**Background:**

A review of research priorities completed by WHO technical units was undertaken. Results of the mapping were recorded in a database that was used to generate analysis and compare research priorities and the different methodological approaches used in their development.

**Methods:**

A total of 116 documents were reviewed for this study. The documents were published between 2002 and 2017 by the technical programmes of WHO headquarters and deposited in the institutional repository, IRIS. Research priorities were extracted from documents into a standard template and mapped to a five-category research cycle type framework defined in the WHO Strategy on Research for Health covering research to describe the research problem, identifying the cause and risk factors, developing solutions and new interventions, understanding the barriers to implementation, and evaluation of the impact of response. Details of the research priority methods were recorded. A database with user interface was created using Microsoft Excel 2010.

**Results:**

A total of 2145 research priorities were extracted from the 116 documents meeting the inclusion criteria. The priorities specifically address 73 diseases/health topics. The document types were 26% Report, 22% WHO Guideline, 26% Research Prioritisation publication and 11% Meeting Notes. The most widely reported method used to identify priorities was expert consultation. Expert consultation was used to identify 86% of the priorities categorised here, with 26% (561) reporting it as the sole method; 52% (1111) explicitly listed a literature review as contributing to the identification of priorities. When the 2145 priorities were categorised across the research cycle framework, the largest portion (43%) addressed implementation challenges. The database is published here under an open access licence.

**Conclusion:**

Comparing research priorities between diseases/health topics requires standardisation and the research cycle type framework is one approach that can be applied across all the health topics found in public health. There is great variation in the use of research priority-setting methodology at WHO Headquarters. Therefore, a standard reporting approach, linked to established good practice, should be an area for future development by the WHO Global Health R&D Observatory. The database reported here can also be used to quickly access and analyse the research priorities for a specific health topic or to compare across a range of health topics.

**Electronic supplementary material:**

The online version of this article (10.1186/s12961-018-0391-0) contains supplementary material, which is available to authorized users.

## Summary

This paper describes how a standard template was used to map the reported health research priorities undertaken by the technical programmes of WHO between 2002 and 2017 and demonstrates the value of standardisation in providing an analysis of a research portfolio as an approach to inform the development of the WHO Global Health R&D Observatory.

## Background

The setting of priorities for health research is an essential part of managing a health system. Prioritisation helps ensure the most effective use of resources (such as research capacity, time and funds) for optimal health impact. While there are a number of published methods describing different approaches for setting priorities for health research, there is no single best practice [1–3].

Consequently, there is a great variation in the approaches used to organise research priority-setting exercises. These range from meetings or surveys that collect expert opinion, to more systematic methods that combine a review of the literature, inclusive Delphi surveys of stakeholders and a recognised method for identifying the priorities against weighted criteria [4]. When it comes to reporting research priorities, the variation in methods greatly expands as there are no standard reporting templates or guidelines (like the Consort tools available for reporting a clinical trial) [5]. For any individual exercise, this may not be a problem, but when the research methods are not described, for example, which stakeholders were involved in the exercise and their role, it is difficult to judge the validity of the priorities reported. The variation also creates a significant barrier to aggregating and analysing research priorities or conducting any comparative work between research strategies across disease areas and public health issues.

Recognising a need to improve the methods for managing and reporting research – the science of how science is conducted –the WHO Global Health R&D Observatory launched a call for papers in 2015 encouraging researchers and institutions to develop new methods to support analysis and priority-setting for health research and development. The Thematic Series on health research and development has the goal to guide future decision-making and priority-setting in this area [6].

In response to that call, this research was undertaken to review research priority-setting undertaken by the technical programmes based at the WHO headquarters in Geneva from 2002 to 2017. The priorities reviewed were those reported in WHO documents (reports, meeting notes and guidelines) that are publicly available through the WHO Institutional Repository for Information Sharing (IRIS) [7]. Our aim was to see if the impact of the variation between reported research priorities can be overcome by a standardised mapping of the priorities against a common framework. The framework used was an adaptation of the cycle of research type first defined in the WHO Strategy on Research for Health and further elaborated in the World Health Report 2013 [8, 9].

Individual priorities, once extracted and placed in a database, could be analysed across all of the WHO health domains. This data could also be used to visualise and compare the research strategies outlined by those priorities. Information from the original documents would also allow documentation of the different methodological approaches used in setting the research priorities.

## Methods

### Data collection

Data was collected by reviewing documents published by WHO technical programmes at the headquarters in Geneva from 2002 to 2017 and contained in the institutional repository, IRIS. Deposition in IRIS ensures the document is free-to-access in a digital archive and is assigned a permanent url, as opposed to the url for a web page where links can break and be lost over time with editing and updates. This ensures users can always locate the original source document. The data collection was undertaken by interns working within TDR, the Special Programme for Research and Training in Tropical Diseases, under the guidance of the first author of this paper in three, 12-week phases (May–July 2013, May–July 2015 and May–July 2018). The types of publication included guidelines, rapid advice guidelines, strategy documents, action plans, fact sheets, roadmaps, blueprints, meeting notes and research prioritisation reports. The search criteria in IRIS searched the full-text of documents containing the phrases ‘research priority’ and/or ‘research priorities’. While many of the WHO publications are available in the six official languages of WHO, this work utilised the English language version of the documents.

The inclusion criteria required that the document was (1) covering global health research priorities identified by technical programmes at the WHO headquarters and published in English; (2) publicly available in the WHO IRIS with a permanent url; (3) an official WHO publication that has been through the internal clearance process and has the WHO logo within the publication; and (4) published between the years 2002 and 2017.

The inclusion criteria were created to produce a manageable dataset of publications that shared the same objectives and that represented official WHO publications concentrating on health research priorities from a global perspective.

The exclusion criteria were (1) documents produced by WHO regional and country offices; (2) publications by other UN agencies or global health research agencies, for example, research funders; and (3) journal articles and systematic reviews. The exclusion criteria were chosen to keep the number of documents under review manageable to answer our question about the value of standardisation with respect to comparing priorities across disease areas.

An initial search of IRIS from 2002 to 2017 retrieved 2840 documents. A review of these 2840 documents identified 280 that merited more in-depth review according to the inclusion criteria. Of these 280 documents, 116 were eventually retained for use in this study.

Short summaries (‘data captures’) were created to transfer the relevant information within each of the 116 documents to a standard template. Identical priorities were reported in two separate documents and therefore there were 115 data captures. The information captured included publication title, publication date, permanent url, disease/health topic, contact (author, editor, etc.), executive summary, key findings/areas needing further research, priority-setting approach/methodology, measurement/ranking criteria, research financing estimates, limitations, other relevant information (pipeline/timeline/feasibility), and key figures and tables.

The data captures were sent to the WHO department representatives and/or authors of the documents for confirmation that their document had been summarised accurately. This sharing of information was accompanied by a request for other relevant documents. In addition, representatives from 34 of the 36 technical programmes at WHO headquarters were interviewed. These interviews often provided additional information regarding the methods and the priorities identified. However, to maintain the integrity of the study, only the information contained in published documents was analysed.

**Fig. 1:**
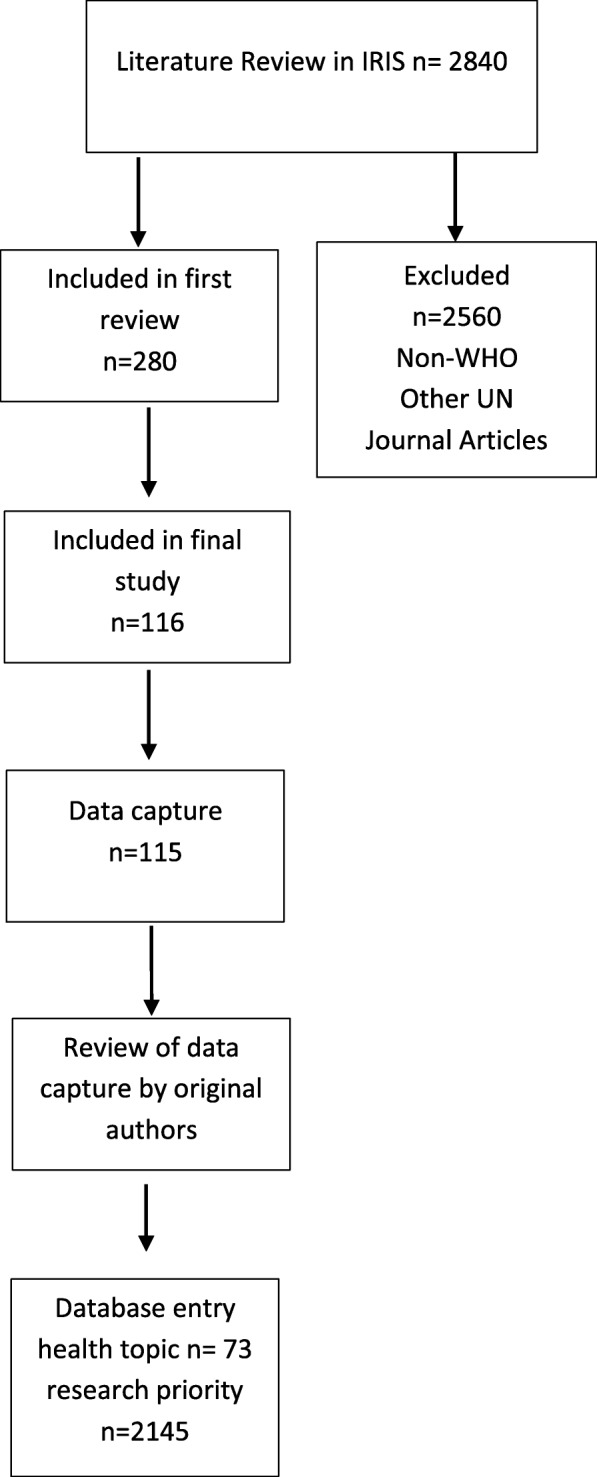
PRISMA study flow diagram

The individual priorities were then extracted and entered into a Microsoft Excel 2010 database to enable data analysis. Phrases and spellings were transcribed as reported in the original document. See PRISMA diagram flow Fig. 1.

### WHO Health Research Priorities database (Additional file 1)

The data were entered into seven integrated worksheets. Table 1 explains the relationship between the worksheets.

**Table 1:** Research priorities database showing priorities from WHO publications (2005–2017) deposited in the WHO Institutional Repository (IRIS)

Tab Colour/Name	Instructions
Definitions	Definitions of methods and criteria included in the database, and definitions for categorising priorities by research area (problem, cause, etc.) adapted from WHO Strategy for Research and Health, 2010.
User Interface	Use dropdown list to compare research areas of specific diseases/health topics with all priorities in the database. The interface also shows the solution-focused priority breakdown, into specific intervention type, for the selected disease/health topic.
Aggregated Database	All information about research priorities and their documents is recorded here. The other sheets access data from this sheet. Clear all filters and set to Select All for analysis of whole dataset. Use filters to analyse by different categorisations. For example, in Column G uncheck Select All and check Guidelines to analyse by Document Type/Guidelines only. The analysis will present on Statistics worksheet. Note: remember to remove filters, i.e. check Select All in all columns to analyse the whole dataset.
WHO Documents Inventory	All documents used in this database are listed here including the permanent url link to the WHO institutional repository (IRIS). To understand the priorities in their proper context users should reference the original publication.
Filter Priorities by Disease	Use to filter by disease/health category and look at the distribution of solution priorities by filtered disease.
Statistics	This displays the analysis of the dataset covering: - Health Topics - Type of document (research prioritisation, report, meeting notes) - Publication date - Prioritisation methodology Note: the analysis will relate to the filters selected in the Aggregated Database.
Solution Statistics	This sheet contains charts that automatically update to show the portion of priorities per research area and of specific interventions for solution priorities.

### Data analysis

The 115 data captures summarising publications containing research priorities were analysed using the Excel database to capture three main categories of data, namely (1) general publication information, (2) prioritisation methodology, and (3) research type in relation to the WHO Strategy on Research for Health.

The database was designed to study the distribution of research priorities across the research cycle of individual diseases/health topic categories. The database also allows analysis of the methodologies used to develop individual research priorities. Each priority has a specific entry number in the database.

General publication information added to the database includes source location, health topic(s) covered, publication date, and type of document (research prioritisation as self-described in title or introduction; report, namely an official WHO publication, but not specifically presented as a research prioritisation publication; or meeting notes, namely unpublished or less formal WHO documents). Research prioritisation documents were official prioritisation reports, while reports were formal WHO reports such as guidelines, action plans and strategy outlines.

Disease/health topic research priorities were narrowed to the most specific category referenced in the publication. For example, both ‘Neglected Tropical Diseases’ and ‘Leishmaniasis’ exist in the database as disease/health topic, but research priorities were categorised as ‘Neglected Tropical Diseases’ if they did not address one of the specific neglected tropical diseases.

The ‘expert consultation’ method of priority-setting was further classified by the composition of the expert group, including academic, medical organisations, international organisations, government, non-government, funding bodies, private sector, and advocacy/stakeholders. The priority ranking criteria used in this study were as follows: public need, magnitude of impact, scientific feasibility, cost, equity, sustainability, curative versus preventative, critical need, and pro-poor.

The methods used to identify research priorities were added to the database. Relevant information on methodology included research prioritisation methods (e.g. expert consultation, literature review, quantitative prioritisation, interviews, Delphi, CHNRI), priority ranking criteria, disease/health topic per priority, and broad and specific research gaps and priorities. For a discussion of research priority methods see Viergever et al. [2].

Priorities were categorised at two levels under a heading of Identified Gaps. Broad research gaps and priorities identified a research area or a research classification (e.g. basic or operational research). The broad gaps and priorities were included only if they grouped together more specific gaps and priorities in the document; therefore, there are a number of blanks in this column. The specific research gaps and priorities were then categorised as a ‘research cycle type’ by mapping against one of the five categories in the research cycle first defined in the WHO Strategy on Research for Health and further elaborated in the World Health Report 2013 [8, 9]. We decided to use this framework as it is simple and applicable to the wide range of public health issues that WHO works with, including infectious and non-communicable diseases, child and maternal health, health systems, environmental health, food safety and many more.

The research cycle categories were adapted and defined in this research as follows:

1. Problem: research to measure the size of the health problem through epidemiology, estimating the burden of disease and other forms of data collection.
2. Cause: research to understand the causal agents, risk factors and determinants of the health issue. May include, for example, study of infection cycle, vectors, role of socioeconomic factors, environment, diet and the interaction of multiple factors.
3. Solution: research to develop new interventions; includes therapeutics, devices and procedures as well as policy interventions, public health campaigns, etc.
4. Implementation: research to translate new interventions into policy and practice and understanding the barriers to delivering known interventions.
5. Evaluation: research to monitor and evaluate the effectiveness or health impact of an intervention or programme.

As research priority-setting often has a focus on the research and development of products (diagnostics, pharmaceuticals and vaccines) and other health interventions, this research cycle type was broken down further to include the following categories: drug, vaccine, diagnostic or screening intervention, vector control, device, basic science, or other/unspecified. An additional three other categories allowed us to record when the research priority was focused on improving an existing intervention (e.g. testing a new combination therapy) or a new intervention and whether there was a specific target population for women or children (paediatric formulations). Each data capture was reviewed by a minimum of two members of the study team and the lead authors/contact point for each document was also asked to verify the data captures. While not all lead authors replied to our request to verify the data captures, of those that did provide their input, not one challenged the premise of the study or the categorisation method. This provided a positive support that our approach was understood and accepted by those technical programmes undertaking research priority-setting.

The database was constructed as a binary code. As each individual priority was categorised across the various Excel columns, a number 1 was placed in each applicable box and a 0 in non-applicable boxes. Using this binary method, number patterns could be analysed for comparison between categories and for summation (quantification) of specific responses. For example, positive responses for each category can easily be summed at the end of a column, while simple Excel equations can identify positive responses across a sheet or various columns. For the intent of this research, the results of selected filters were set-up to display on the Statistics worksheet. Source documents are archived in the WHO repository IRIS with a permanent url so a user can always locate the priority within the context of the original document from where it was extracted.

## Results

During the course of this study, a database was developed that enables a wide range of analysis. This database has been published under an open access creative commons licence. As a result, anyone can access and use our tool for their analysis. A sample of our research outputs are highlighted below, with more analysis being accessible via the database’s *Statistics* worksheet.

From the 116 documents meeting the inclusion criteria of our study, 2145 research priorities were extracted, addressing 73 diseases/health topics. The document type break-down was as follows: 26% report, 22% WHO guidelines, 26% research prioritisation publication and 11% meeting notes.

The majority of the extracted research priorities (72%) came from documents published in 2012 or later. This might reflect the launch date of IRIS in July 2012 (see limitations above). Documents from 2017 provided the most priorities of any year studied, adding 470 priorities (22%) to this dataset.

### Research prioritisation methods

**Fig. 2:**
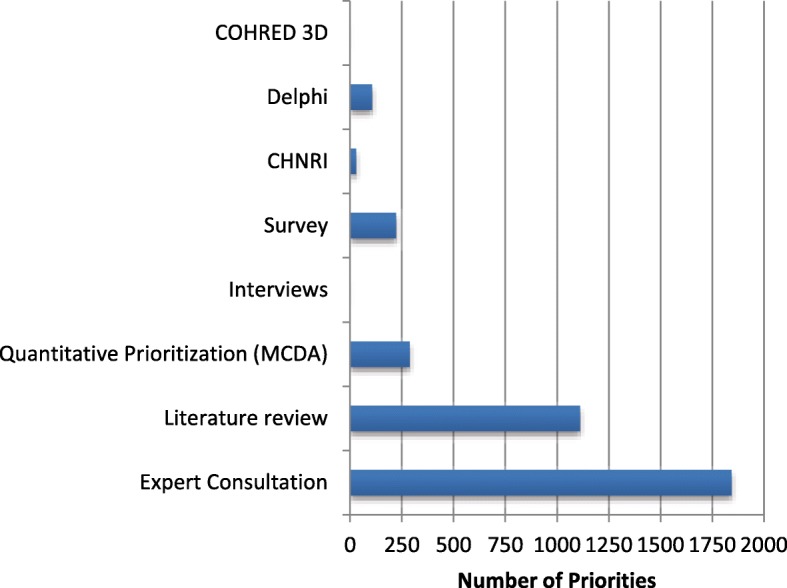
The distribution of research prioritisation methods extracted from WHO publications published 2002–2017. *COHRED 3D* Council on Health Research and Development 3D Combined Matrix Approach. *Delphi* a method iterative surveying to identify consensus often used in assessing future needs and governmental foresight exercises. *CHNRI* Child Health and Nutrition Research Initiative to enable a ranking of priorities by weighted criteria. *Survey* structured opinion gathering with a defined set of stakeholders. *Interviews* in-depth interviews with a selection of stakeholders. *Quantitative prioritisation (MCDA)* Multi-criteria decision analysis to enable a ranking of priorities by weighted criteria. *Literature review* using a systematic review method. *Expert consultation* commonly a face-to-face meeting often, but not always, to review the findings from other priority-setting exercises.

Of the priorities in this dataset, 10% (204 out of 2145) had no research prioritisation method described in the publication. The other methods that were used are shown in Fig. 2. This breakdown is also available in the database on the *Statistics* Worksheet. The filter function of Excel can be adjusted and used in combinations to undertake alternative analysis. For example, an analysis of one or several health topic(s), can be made by checking the correlated filter(s) on the aggregated datasheet.

Of the 2145 priorities in this dataset, few were generated using a published research prioritisation framework, such as Child Health and Nutrition Research Initiative (*n* = 30), Multiple Criteria Decision Analysis (*n* = 132) or Delphi (*n* = 107). None of the priorities were generated using the COHRED 3D method.

The most common method used to identify priorities was expert consultation. Expert consultation was used in 86% of the priorities categorised here, with 26% (561) of those reporting expert consultation reporting it as the sole prioritisation method. While 1111 (52%) of the priorities reviewed described the use of a literature review in priority identification, all 1111 combined the literature review with expert consultation. The expert consultation results are contained in 61 documents, of which 38 are WHO Guidelines including one Rapid Advice Guideline. Of these, only the Guideline documents referenced the use of the GRADE method to assess the quality of the evidence used. GRADE (Grading of Recommendations, Assessment, Development and Evaluation) is the standard used by WHO when developing guidelines [10].

Almost 70% (2110) of the identified research priorities were developed without using any additional criteria to rank the priorities with respect to potential public health impact, feasibility of undertaking the research or cost. In fact, some kind of cost estimate was only mentioned in 10 of the 116 documents (all documents mentioned in the paper are listed in IRIS; http://apps.who.int/iris/), representing 321 priorities. Only a few documents, such as the Network of WHO Collaborating Centres for Trachoma: Second Meeting Report (2016), supplied detailed finance estimates for their research priorities, but the majority or reports did not.

#### Variation in reporting research priorities

The approach to reporting the research priorities varied greatly. The number of priorities per document also varied, from more than 100 priorities in the ‘WHO Public Health Research Agenda for Influenza’ (2017 update) to fewer than 10 in ‘Transgender People and HIV’ (2015). However, this is very context specific and so a document setting out a research roadmap would be expected to detail more priorities than a broader report on a health topic where research is only one part of the review.

Even after excluding documents that only included broad research priorities, the degree of specificity in describing a priority also varied greatly. Priorities ranged from general statements of need, such as “*Discovery and development of new trematocidal drugs*,” to very specific requests, like “*Focused research and development is required to produce live replication-competent vaccines for oral and other routes, which are more effective in wildlife primary hosts such as raccoons, mongooses and skunks and safe for non-target species, including humans*” (Rabies).

### Mapping of priorities to the research cycle (type of research)

**Fig. 3:**
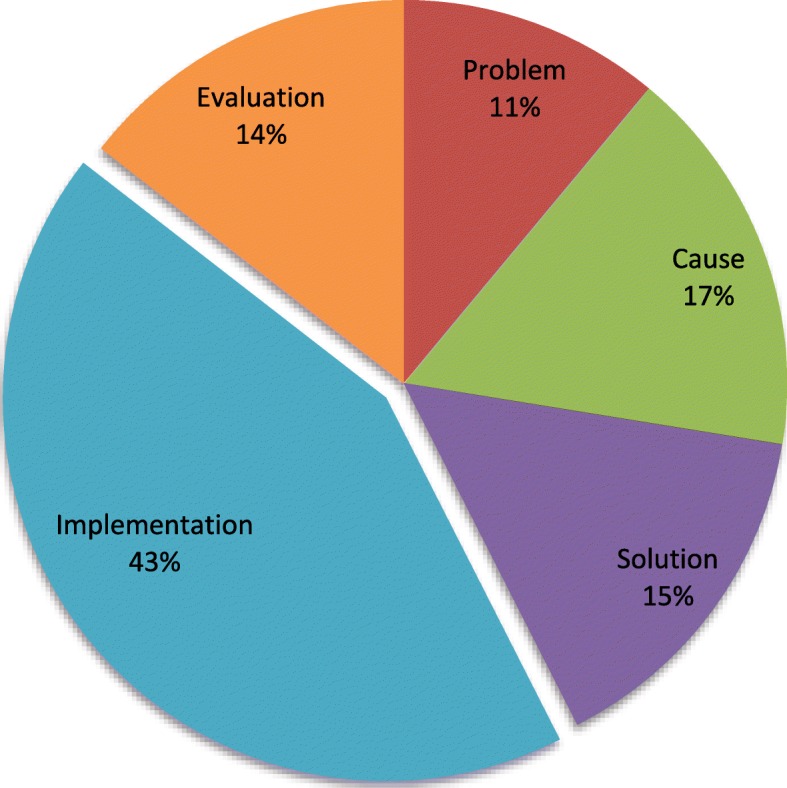
Distribution of research priorities by research type (*n* = 2145). Extracted from WHO publications published 2002–2017. The five research type categories were adapted and defined here as: 1. Problem: research to measure the size of the health problem through epidemiology, estimating the burden of disease and other forms of data collection. 2. Cause: research to understand the causal agents, risk factors and determinants of the health issue. May include, for example, study of infection cycle, vectors, role of socioeconomic factors, environment, diet and the interaction of multiple factors. 3. Solution: research to develop new interventions. Includes therapeutics, devices and procedures but also includes policy interventions, public health campaigns, etc. 4. Implementation: research to translate new interventions into policy and practice and understanding the barriers to delivering known interventions. 5. Evaluation: research to monitor and evaluate the effectiveness or health impact of an intervention or programme

Of the 2145 priorities categorised across the research cycle as addressing a problem, cause, solution, implementation or evaluation, the largest portion (43%) addressed an implementation need (Fig. 3). Examples of how research priorities were classified by research cycle type include the following:

Problem (*n* = 237, 11%)

Examples: Define the population at risk and the global burden of the disease accurately (Leishmaniasis), document the burden of cryptosporidiosis in young children in developing countries (Cryptosporidiosis), the need for low-cost effective surveillance mechanisms for leptospirosis in endemic countries (Leptospirosis).

Cause (*n* = 354, 17%)

Examples: Assess, by further investigation, the significance of serotypes and genotype switch and potential for outbreaks in areas endemic for Dengue, assess the contribution of systematic non-compliant persons as well as pregnant/lactating women and under five-year-olds to the maintenance of transmission, and study the possibilities of including the former in control programmes (Helminths).

Solution (*n* = 321, 15%)

Examples: To develop an anti-toxin for use as a vaccine (Buruli Ulcer). Development of a non-invasive POC test to detect and discriminate first and second stage HAT (Human African Trypanosomiasis).

Implementation (*n* = 922, 43%)

Examples: Develop means to train health professionals on all aspects of cancer control, including leadership and management of cancer control programmes with a public health approach at the national and district levels (Cancer). Develop messages effective in overcoming misinformation spread by tobacco companies, building and strengthening social norms against tobacco, and building support for tobacco control policies and programmes (Tobacco). Improved criteria for identifying low-birth-weight (LBW) infants who need to be cared for in a hospital.

Evaluation (*n* = 311, 14%)

Examples: Development of appropriate and gender-sensitive tools and methods is needed to assess the health and socioeconomic impact of control programmes on individuals and households (Asian Schistosomiasis). What is the effectiveness of zinc supplementation on the outcome and incidence of diarrhoea in the community? (Childhood diarrhoea).

### Looking at solutions

Of the 338 priorities that address solutions, some address more than one health technology, with the largest portion (31%) targeting diagnostic or screening solutions; 18% address drug solutions and 15% address basic science. Of the solution priorities which specified whether the solution focused on developing a new or improving an existing solution, 51% (115 of 338) aimed to develop a new solution and 34% to improve an existing solution.

The gender and paediatric emphasis in solution research priorities was also examined; 11% of solutions were targeted at the paediatric population and 4% were targeted at women.

### Priorities by health topic/disease topic

The top 10 disease/health topics by the number of priorities in published reports were HIV/AIDS (*n* = 252), tuberculosis (*n* = 239), nutrition (*n* = 117), malaria (*n* = 109), influenza (*n* = 101), maternal and child health (*n* = 96), zoonoses (*n* = 83), Zika (*n* = 64), trachoma (*n* = 52) and leishmaniasis (*n* = 52).

**Fig. 4:**
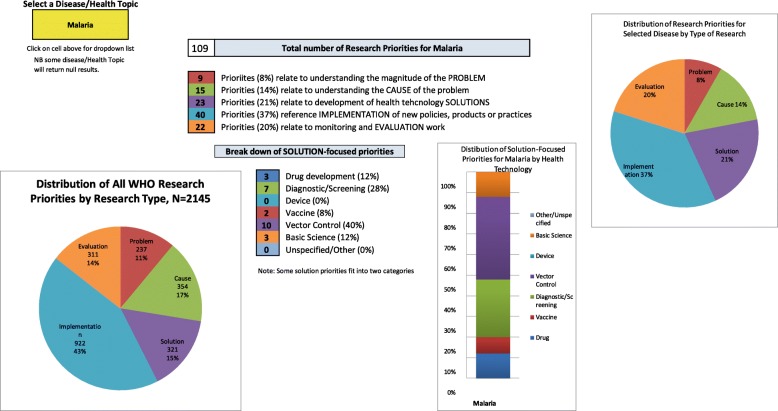
Example of output from user Interface showing distribution of the 109 research priorities identified for malaria by WHO technical programmes in documents published between 2002 and 2017. Readers are advised to access the database directly to view the outputs at a higher resolution and create their own outputs

The *Interface* worksheet in the database allows the user to select a specific health topic, gain an overview of the research priorities in that topic and compare those results to the whole dataset. An example of this is show in Fig. 4. This figure shows a breakdown of the 109 research priorities identified for malaria in WHO technical programme documents published between 2002 and 2017. The malaria results show that the research strategy (by distribution of priority across the research cycle type framework) is similar to the overall research strategy of WHO. Both have a majority of priorities in the area of implementation research – WHO (43% of priorities identified as implementation) and malaria (39% of priorities identified as implementation). The distribution of research priorities for malaria within the Solution Research Type breakdown further to show vector control (40% of priorities) and diagnostics/screening (28% of priorities).

### Comparison between diseases/health topic

**Fig. 5:**
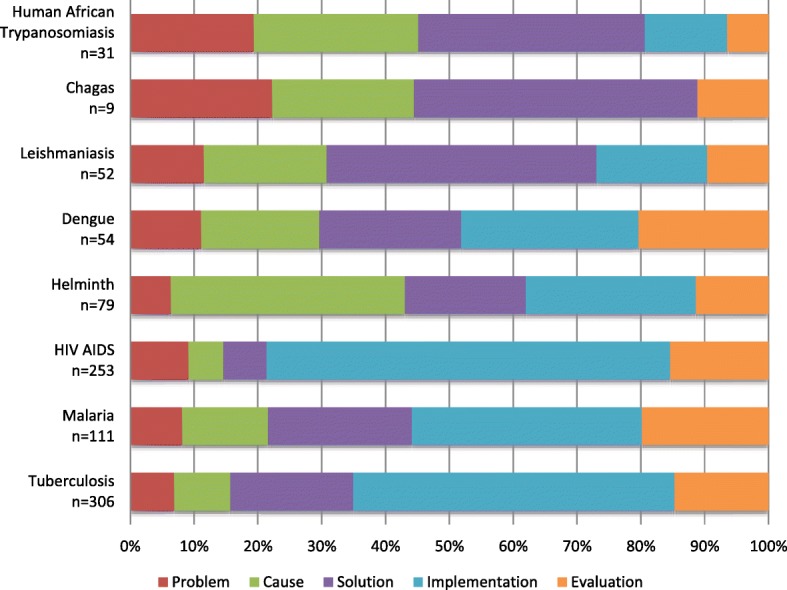
Distribution of research priorities comparing eight infectious diseases. Priorities extracted from WHO publications between 2002 and 2017

**Fig. 6:**
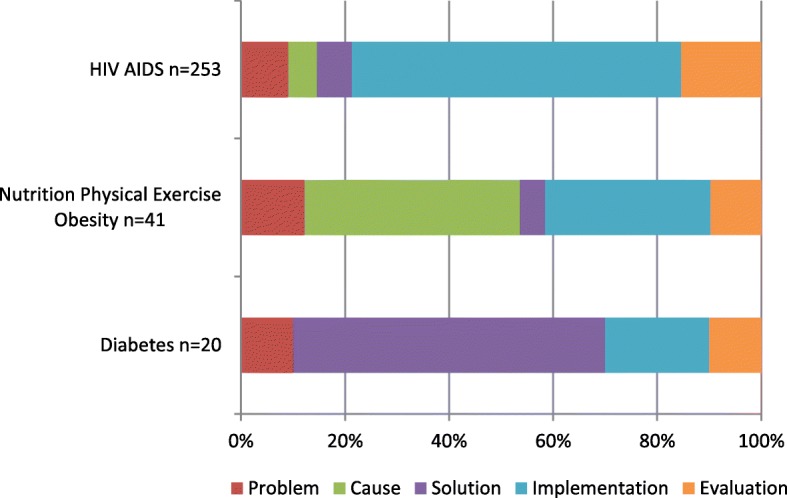
Distribution of research priorities for HIV/AIDS compared with nutrition and physical exercise and diabetes extracted from WHO publications published between 2002 and 2017. The graph shows the different emphasis or priorities across the research cycle

One of the unique analyses this study has enabled is a visual representation of the different research strategies, as expressed by research priorities, when mapped to a standard template. Figure 5 shows a comparison between eight infectious diseases, wherein tuberculosis and HIV/AIDS have a majority of research priorities focussed on implementation needs, while human African trypanosomiasis, Chagas and leishmaniasis have priorities across the spectrum of research, with a stronger focus on solution development. This variation in research strategy is more marked when a comparison is made between infectious and non-infectious diseases (Fig. 6).

**Fig. 7:**
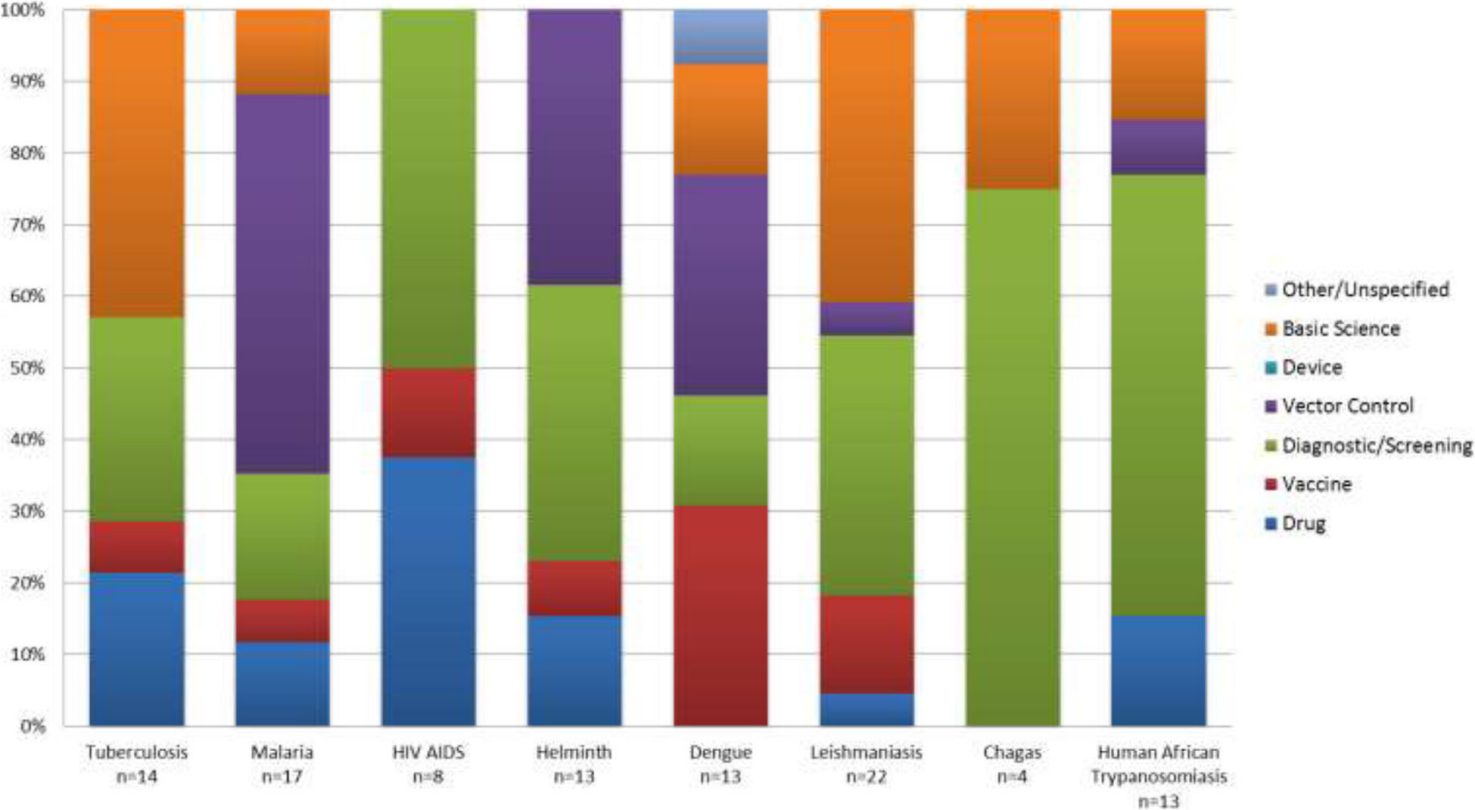
A comparison of research priorities with a focus on solution extracted from WHO publications published between 2002 and 2017

This comparison can also be undertaken between the diseases/health topics with respect to the priorities for the solution research type (interventions, health technologies, etc.). Figure 7 shows the different solution priorities identified for the same eight infectious diseases and reveals very different strategic shapes.

## Discussion

### Study strengths

The results of this research support the original hypothesis that, in the absence of common approaches, research priorities can be summarised and mapped against a standard template. The research cycle type template from the WHO Strategy on Research for health can be applied to all the health sectors in which WHO is engaged and the scope broadened to include other agencies active in health research. The fact that data collection was spread over three time periods by three different members of the study group and yet consistency was maintained, suggests the application of this framework is simple and easy.

The resulting database enables a comparison to be made between the different sets of priorities and allows the creation of a visual representation across the research portfolios. This provides a new tool for informing discussions about research priorities, particularly with respect to a comparison of the strategic shape of those priorities. That shape is expressed as the proportion of priorities spread between understanding the scale of a health problem, identifying its cause(s), discovering solutions, implementing those solutions, and evaluating the impact. We see for WHO as a whole, and for the major infectious diseases, that implementation research is a high priority. Yet, when the funding available for research is mapped, we see there is a funding gap with respect to implementation as highlighted in a recent publication (An Analysis of Funding—From Basic Research and Product Development to Research for Implementation [11]).

The database is published here under an open access licence with all the data and user instructions and can be used and adapted by others to undertake their own analysis and improve the approach to comparing priorities reported here.

### Limitations of the study

This study is deliberately bound by inclusion criteria. The intent is to create a manageable subset of data related to WHO research prioritisation activities. For example, regional and country office and journal publications are excluded. As such, this work is not a comprehensive list of all research priorities identified by WHO. The IRIS resource was first published in July 2012. While great efforts have been made by IRIS to capture all WHO publications going back to 1948, there might well be past publications that have yet to be digitally archived, including documents published during our study’s timeframe of 2002–2017.

The analysis of research priority methods is also limited to those approaches used by WHO and recorded in published reports. These methods might not be representative of the field as a whole. Additionally, activities such as the creation of an expert steering group might have been undertaken, but if they are not reported alongside the priorities themselves then it is the equivalent of only reporting results in an academic paper while omitting the methods section.

The use of research cycle type as a categorisation of research purpose is new and requires judgement. The data capture took place over three separate periods with different individuals, interns at TDR, collating the data. However, the principle author provided continuity and the individuals were required to review previous work to ensure they aligned themselves with the approach. While not all the data captures were confirmed by the lead authors of the original documents, of those that did provide input, none challenged the premise of the study or the categorisations we had made. This supported our assumption that the research cycle framework was suited to mapping research priorities across the entire public health spectrum.

It is important to note that the volume or number of research priorities per disease area is not a measure of importance. However, the number of priorities does broadly relate to scale of research activity undertaken by a technical department in an area and that is supported by previously reported surveys on research activity at WHO headquarters [12]. The method is labour intensive and seriously hampered by the wide variety of research methods used and the format for reporting them. Developing a standard reporting approach for WHO would be one of the areas that the Global Health R&D Observatory should explore. An initial template of good practice in research priority-setting already exists [2] and could form the basis of such guidance.

However, it is less certain that a single standard methodology for setting research priorities across all health topics could be developed. For example, a systematic review is strongly recommended as the starting point for any research. Yet, a systematic review, even where quality evidence exists, can only identify knowledge gaps [13]. There remains a need to use multi-dimensional inputs from stakeholders and feasibility studies to turn identified gaps into priorities. Therefore, priority-setting methodology would need to reflect the context and standards and should be developed by disease/health topic under the principles of a standard reporting framework.

## Conclusions

Comparing research priorities between diseases/health topics requires standardisation and the research cycle by type of research framework is one approach that can be applied across all the health topics in public health. There is great variation in the use of research priority-setting methodology at WHO headquarters, and while a standard method for all research in all health areas might be difficult to achieve, a standard reporting approach, linked to established good practice, should be an area the WHO Global Health R&D Observatory works to develop. The database reported here can also be used to quickly access and analyse the research priorities for a specific health topic or compare across a range of health topics. While the method was only applied to health research priorities identified by WHO technical programmes, the method is adaptable enough that it could also be used by other agencies in global health and could support efforts to achieve greater harmonisation in global public health research. The need to apply the checklist of good practice in priority-setting identified by Viergever et al. [2] remains as pressing today as when it was first reported in 2010.

## Additional file

Additional file 1: Database Analysis of Health Research Priorities. (XLSX 1264 kb)
